# Confirmed Myocarditis-Myositis Overlap Without Myasthenia Gravis: Evaluating for Triple M Syndrome Following Combined Enfortumab Vedotin and Pembrolizumab Therapy

**DOI:** 10.7759/cureus.116849

**Published:** 2026-09-24

**Authors:** Bryan Ashong, Yash Ranga

**Affiliations:** 1 Anesthesia, University of South Florida Morsani College of Medicine, Tampa, USA; 2 Internal Medicine, Jawaharlal Nehru Medical College, Ajmer, IND

**Keywords:** acetylcholine receptor antibodies, chemotherapy induced adverse effects, drug induced transaminitis, hepatic damage, hepatopathy, immune-checkpoint inhibitor adverse effects, immune checkpoint inhibitor-associated myocarditis, immune checkpoint inhibitor-associated myositis, triple m syndrome, uro oncology

## Abstract

A 64-year-old male with Stage III urothelial carcinoma of the right ureter presented to the emergency department 3 weeks after a single cycle of combined enfortumab vedotin and pembrolizumab with chest tightness, neck weakness, drop head, and myalgias. Laboratory findings included markedly elevated high-sensitivity troponin I, creatine phosphokinase, lactate dehydrogenase, and transaminases. Electrocardiography showed anterolateral ST depression and left anterior fascicular block, which was new as compared to a prior tracing. He was managed in the cardiovascular intensive care unit. Cardiac catheterization revealed concurrent 90% mid-left anterior descending artery stenosis, treated with drug-eluting stent placement. Endomyocardial biopsy confirmed focal moderate lymphocytic myocarditis. Cardiac MRI demonstrated mildly reduced biventricular function and a non-ischemic pattern of late gadolinium enhancement consistent with myocarditis sequelae. Given the overlapping multisystem presentation, a multidisciplinary team (cardiology, neurology, and oncology) elected to treat with high-dose methylprednisolone (1 g QOD x 5 days), the guideline-recommended first-line therapy for ICI-associated myocarditis, and intravenous immunoglobulin (400 mg/kg QOD x 5 days), with biochemical improvement. IVIG was added given clinical concern for possible neuromuscular junction involvement in the setting of concurrent myositis and possible overlap syndrome, reflecting a precautionary approach to a potentially rapidly progressive presentation. Myasthenia gravis was considered but not confirmed: repeated neurological examinations demonstrated no fatigable weakness, and serology was negative for acetylcholine receptor binding and muscle-specific kinase antibodies. Both implicated agents were permanently discontinued. This case illustrates the spectrum and severity of multisystem immune-related toxicity from combined antibody-drug conjugate and immune checkpoint inhibitor therapy.

## Introduction

The combination of enfortumab vedotin, a nectin-4-directed antibody-drug conjugate, and pembrolizumab, a programmed death-1 immune checkpoint inhibitor, has demonstrated significant efficacy in locally advanced and metastatic urothelial carcinoma and has been studied in the adjuvant setting for high-risk disease. Pembrolizumab is well established to cause immune-related adverse events through checkpoint inhibition [1,2], while enfortumab vedotin carries its own distinct toxicity profile, including cutaneous reactions, peripheral neuropathy, and hyperglycemia. Whether the combination confers an increased risk of immune-related adverse events beyond that expected from pembrolizumab alone remains incompletely characterized.

Immune checkpoint inhibitor-associated myocarditis is among the most feared immune-related adverse events, with reported mortality of 25-50% [3]. A constellation of concurrent myocarditis, myositis, and myasthenia gravis, referred to as Triple M syndrome, has been described and carries an estimated mortality approaching 60%, thought to arise from shared antigenic targets in cardiac muscle, skeletal muscle, and at the neuromuscular junction [4]. This case describes a patient who developed severe multisystem immune-related toxicity, including confirmed myocarditis and myositis following a single cycle of combined enfortumab vedotin and pembrolizumab, with a clinical suspicion for Triple M syndrome in which myasthenia gravis could not be definitively confirmed.

## Case presentation

A 64-year-old man with a history of coronary artery disease, hypertension, hyperlipidemia, obstructive sleep apnea, and Stage III (pT3N0M0) invasive high-grade urothelial carcinoma of the right ureter with carcinoma in situ at the distal margin (status post right ureterectomy) was commenced on adjuvant combined enfortumab vedotin and pembrolizumab therapy. Within days of his first cycle, he reported expected side effects including constipation and body aches. These early symptoms were not initially escalated to his oncology team.

Approximately three weeks after the infusion, he developed progressive chest tightness, neck extensor weakness, drop head, and generalized myalgias. His oncologist discontinued both agents and referred him to the emergency department for the evaluation of suspected myocarditis [5]. He reported that the chest tightness was partially alleviated by leaning forward and came and went in character. He had self-administered 20 mg of prednisone and 1 mg of lorazepam prior to arrival. Vital signs were stable on presentation. He denied shortness of breath, palpitations, syncope, peripheral edema, or rash. There was no history of smoking, alcohol, or illicit drug use. Family history was notable for hypertension in both parents.

Physical examination in the cardiovascular intensive care unit demonstrated an alert, oriented, and ill-appearing gentleman. Cardiac examination revealed a regular rate and rhythm with clear first and second heart sounds. Lung fields were clear bilaterally. Neurological examination was grossly intact with cranial nerves II through XII intact, and no fatigable weakness elicited on repeated assessment. The patient had full neck and extremity range of motion with 5/5 strength in all four extremities. No ptosis or bulbar dysfunction was noted. Abdomen was non-tender with bowel sounds present.

Initial laboratory evaluation was notable for high-sensitivity troponin I 568 ng/L (reference <35.0 ng/L), creatine phosphokinase 3,332 U/L, lactate dehydrogenase 730 U/L, pro-B-type natriuretic peptide 165 pg/mL, erythrocyte sedimentation rate 37 mm/hr, C-reactive protein 0.18 mg/dL, and white blood cell count 18.87 × 10⁹/L. Hemoglobin was 12.9 g/dL and creatinine 0.80 mg/dL. Thyroid function testing (TSH, free T4), glucose, and HbA1C were all within normal limits.

Transaminases were markedly elevated at presentation: AST 332 U/L (reference 5-34 U/L) and ALT 335 U/L (reference 0-45 U/L). Both AST and ALT trended downward over the course of the hospitalization in response to immunosuppressive therapy (Table 1). However, alkaline phosphatase, gamma-glutamyl transferase (GGT), total bilirubin, and INR remained within normal limits.

**Table 1:** Serial liver function tests AST: aspartate aminotransferase; ALT: alanine aminotransferase

Date	Time	AST U/L (5–34)	ALT U/L (0–45)
Day 1	11:09	332 ↑	335 ↑
Day 1	22:31	292 ↑	305 ↑
Day 2	05:33	225 ↑	251 ↑
Day 2	12:07	186 ↑	251 ↑
Day 2	18:03	179 ↑	240 ↑
Day 3	02:15	142 ↑	229 ↑
Day 3	09:53	136 ↑	232 ↑
Day 3	13:35	124 ↑	225 ↑
Day 3	17:43	118 ↑	219 ↑
Day 4	02:12	94 ↑	197 ↑
Day 4	04:45	86 ↑	196 ↑
Day 4	12:41	83 ↑	203 ↑
Day 4	16:56	67 ↑	173 ↑
Day 4	23:39	69 ↑	164 ↑
Day 5	02:52	67 ↑	154 ↑
Day 5	11:59	108 ↑	172 ↑
Day 5	18:32	105 ↑	169 ↑

Serial high-sensitivity troponin I values demonstrated a biphasic pattern, with an initial presentation value of 568 ng/L, an early post-catheterization nadir of 254 ng/L, a post-procedural peak of 871 ng/L on the third hospital day, and a subsequent sustained downtrend to 303 ng/L by hospital day six (Table 2).

**Table 2:** Serial high-sensitivity troponin I values

Date	Time	Troponin I hs (ng/L)	Direction
Day 1	11:09	568	—
Day 1	15:16	507	↓
Day 1	22:31	472	↓
Day 2	05:34	254	↓
Day 2	18:03	538	↑ post-PCI
Day 3	02:15	871	↑ peak
Day 3	09:53	767	↓
Day 3	17:43	856	↑
Day 4	02:12	613	↓
Day 4	09:00	443	↓
Day 4	16:56	468	↑
Day 4	23:39	548	↑
Day 5	11:59	503	↓
Day 6	00:15	303	↓

Electrocardiography on admission demonstrated normal sinus rhythm with anterolateral ST depression and left anterior fascicular block (Figure 1). Critically, a prior electrocardiogram performed three months before admission had a normal tracing, confirming that the fascicular block and ST changes were new findings attributable to the current illness (Figure 2).

**Figure 1:**
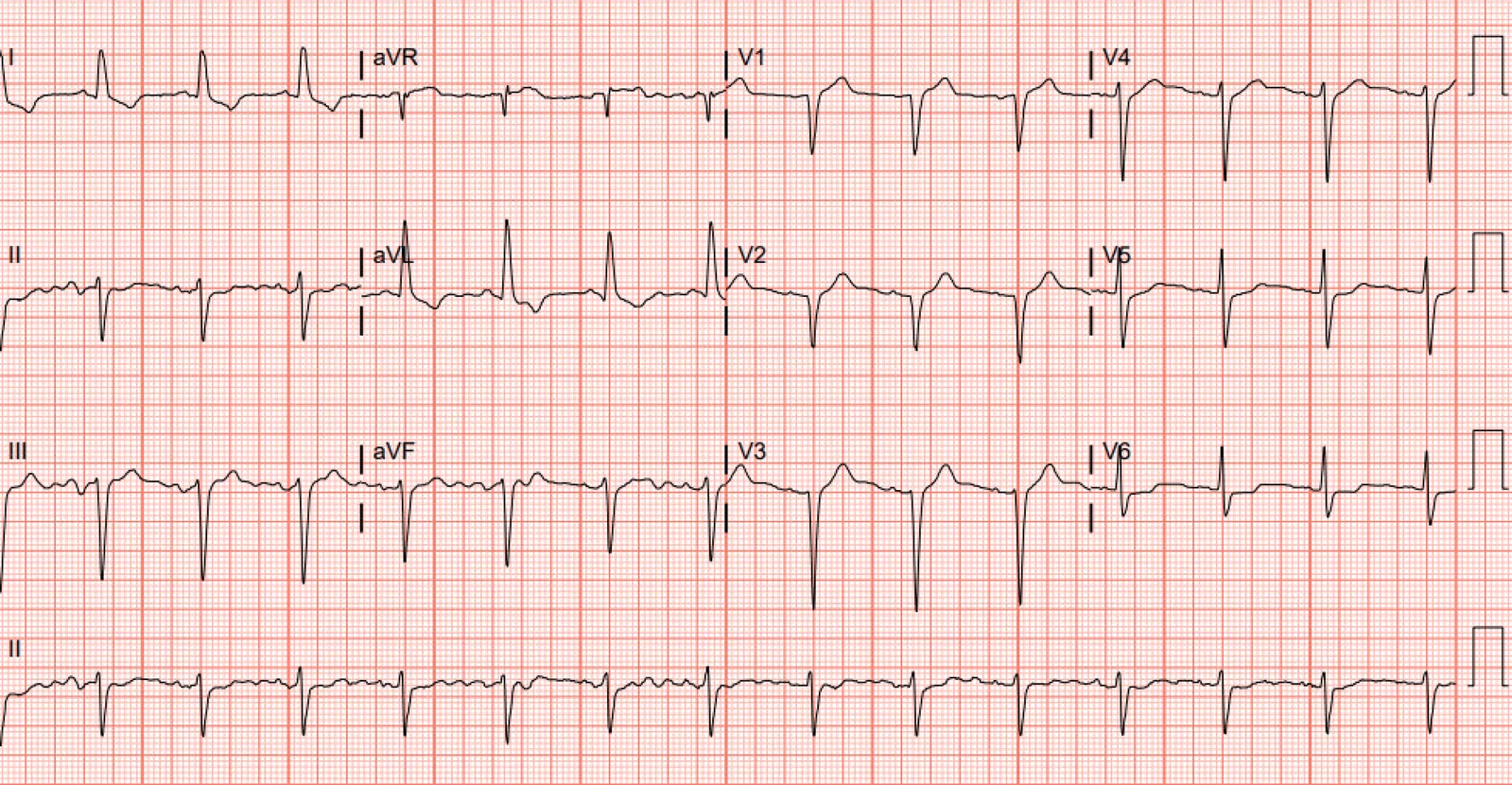
Admission ECG with normal sinus rhythm, mild anterolateral ST depression, and left anterior fascicular block

**Figure 2:**
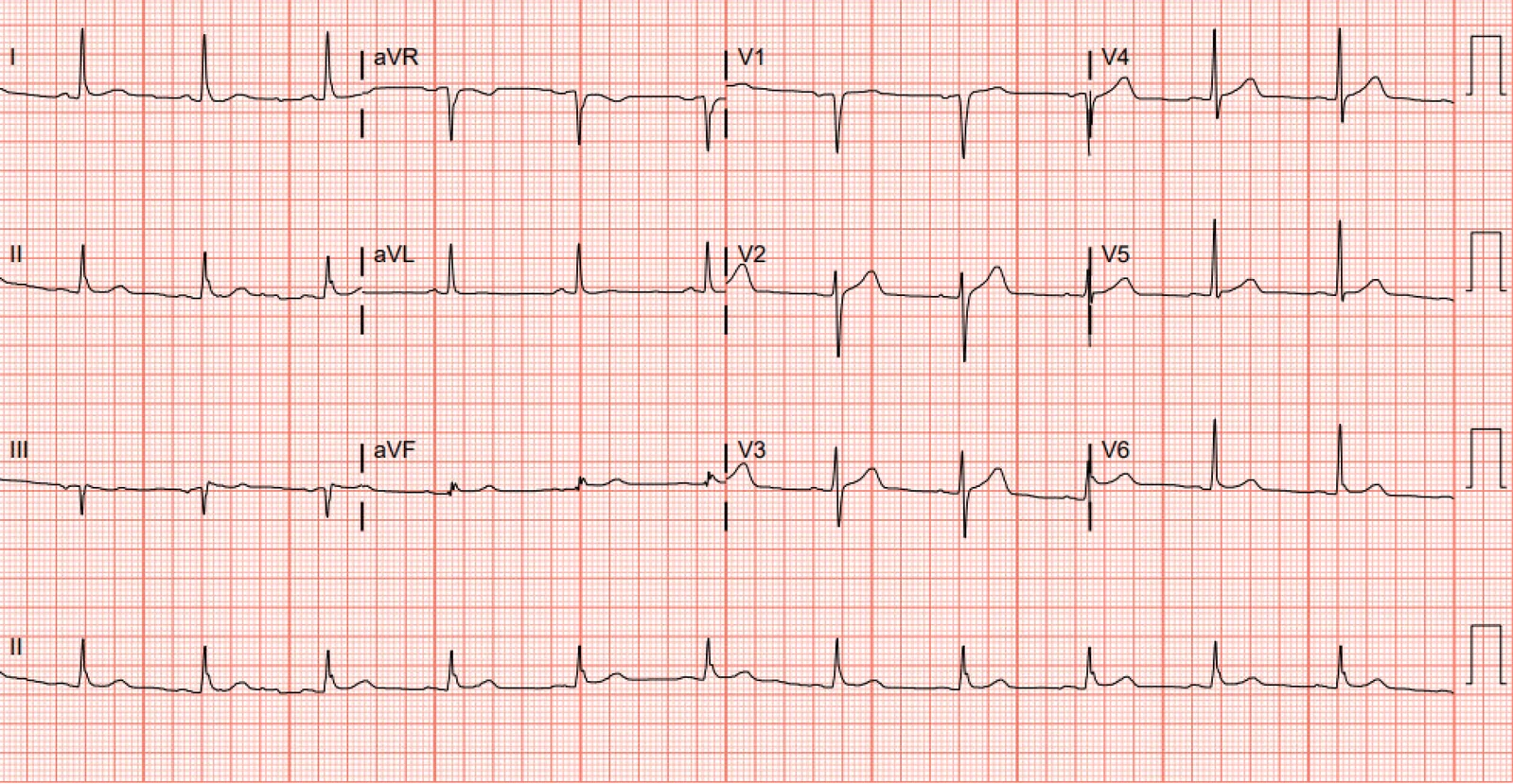
Prior EKG from three months before admission demonstrating normal sinus rhythm and normal axis

Transthoracic echocardiography demonstrated a left ventricular ejection fraction of 55-60% with mild aortic stenosis (Figure 3); global longitudinal strain imaging was attempted but was not interpretable due to suboptimal image quality. Cardiac MRI subsequently revealed a mildly reduced left ventricular ejection fraction of 46% with hypokinesis of the mid-anterior septum and distal septal wall, and a mildly reduced right ventricular ejection fraction of 36%. Given the limited echocardiographic acoustic windows and the absence of interpretable strain data, the discrepancy between echocardiographic and cardiovascular magnetic resonance imaging (CMR)-derived LVEF likely reflects differences in image quality and quantification method rather than a true physiologic change. The left atrium was severely dilated (indexed volume 40 mL/m²), providing structural context for the atrial fibrillation that developed during the admission. Faint mid-myocardial late gadolinium enhancement was identified in the basal inferolateral wall (Figure 4), described as non-ischemic and possibly representing sequela of myocarditis [6]. No late gadolinium enhancement suggestive of left anterior descending artery territory infarct was identified. A follow-up cardiac MRI in three to six months was recommended. All of the lab values are summarized in Table 3.

**Figure 3:**
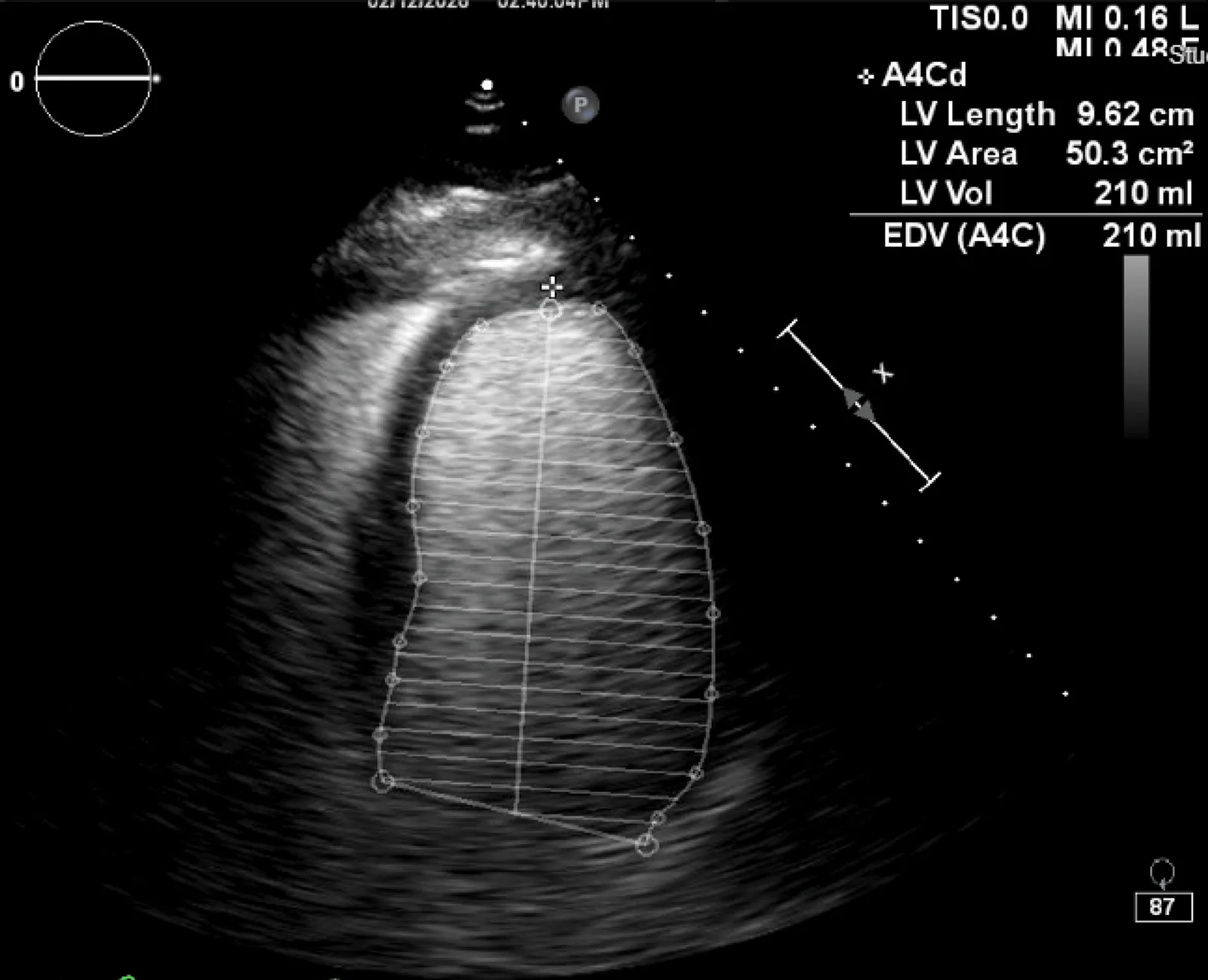
Transthoracic echocardiogram in apical four-chamber view Definity contrast was administered to enhance regional wall motion assessment and opacify the LV. The left ventricle was visualized and determined to have normal ejection fraction.

**Figure 4:**
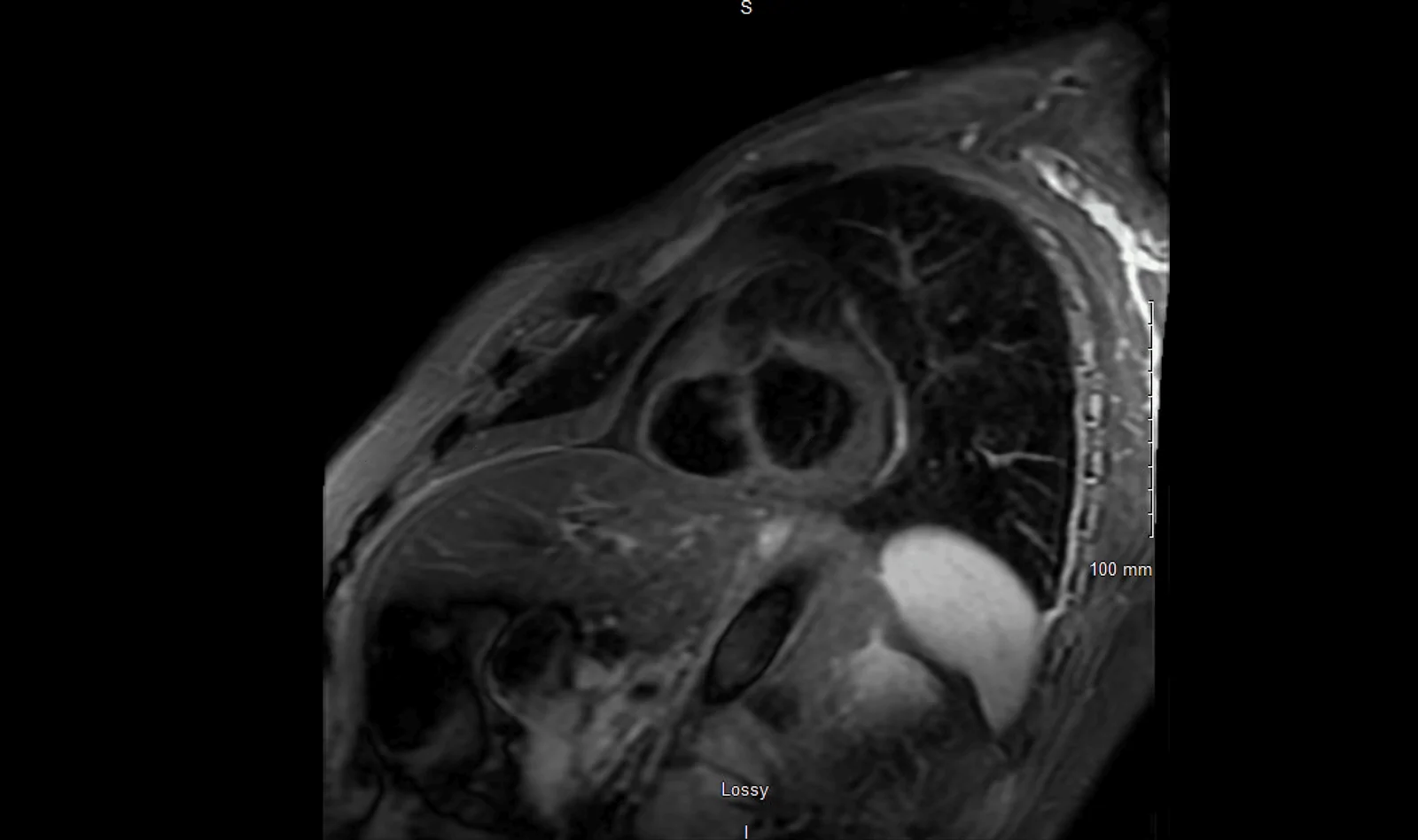
Cardiac MRI demonstrating faint focus of mid-myocardial late gadolinium enhancement involving the basal inferolateral wall

**Table 3:** Summarized lab values CPK: creatine phosphokinase; LDH: lactate dehydrogenase; Pro-BNP: N-terminal pro-B-type natriuretic peptide; AST: aspartate aminotransferase; ALT: alanine aminotransferase; ESR: erythrocyte sedimentation rate; CRP: C-reactive protein

Parameter	Value (Peak/Initial)	Reference Range	Interpretation
High-Sensitivity Troponin I	568 ng/L (peak 871)	<35 ng/L	Markedly elevated – consistent with myocardial injury/myocarditis
CPK	3,332 U/L	30–200 U/L	Severe elevation – consistent with myositis
LDH	730 U/L	140–280 U/L	Elevated – tissue injury (muscle/cardiac)
Pro-BNP	165 pg/mL	<125 pg/mL	Mild elevation – cardiac strain
AST	332 U/L	5–34 U/L	Markedly elevated – hepatocellular injury
ALT	335 U/L	0–45 U/L	Markedly elevated – hepatocellular injury
ESR	37 mm/hr	<20 mm/hr	Elevated – inflammatory state
CRP	0.18 mg/dL	<0.5 mg/dL	Within normal limits
White Blood Cell Count	18.87 × 10⁹/L	4–11 × 10⁹/L	Leukocytosis
Hemoglobin	12.9 g/dL	13–17 g/dL	Mildly low
Creatinine	0.80 mg/dL	0.7–1.3 mg/dL	Normal renal function
Acetylcholine Receptor Antibody	<0.30 nmol/L	Negative	Negative
MuSK Antibody	Negative	Negative	Negative

Right and left heart catheterization on the second hospital day demonstrated 90% de novo stenosis of the mid left anterior descending artery (15 mm length, American College of Cardiology (ACC)/American Heart Association (AHA) Type C lesion, Thrombolysis In Myocardial Infarction (TIMI) 3 flow pre-intervention), successfully treated with a 2.5 × 15 mm Resolute Onyx drug-eluting stent post-dilated with a 3.5 mm balloon, achieving 0% residual stenosis with TIMI 3 flow. Right heart hemodynamics were: right atrial pressure 6 mmHg, pulmonary artery pressure 24/12 mmHg, wedge pressure 12 mmHg, systemic vascular resistance 850 dynes·sec/cm⁵, pulmonary vascular resistance 40 dynes·sec/cm⁵, cardiac index 3.64 L/min/m², and cardiac output 8 L/min. Endomyocardial biopsy demonstrated focal moderate lymphocytic myocarditis with associated myocyte necrosis, considered likely related to immune checkpoint inhibitor therapy (Figure 5). This histopathologic finding, together with the elevated troponin, new conduction abnormalities, and CMR findings, supported a clinical diagnosis of ICI-associated myocarditis; formal categorization using the IC-OS consensus framework was not applied during the clinical workup. CT pulmonary angiography showed no acute abnormalities (Figure 6).

**Figure 5:**
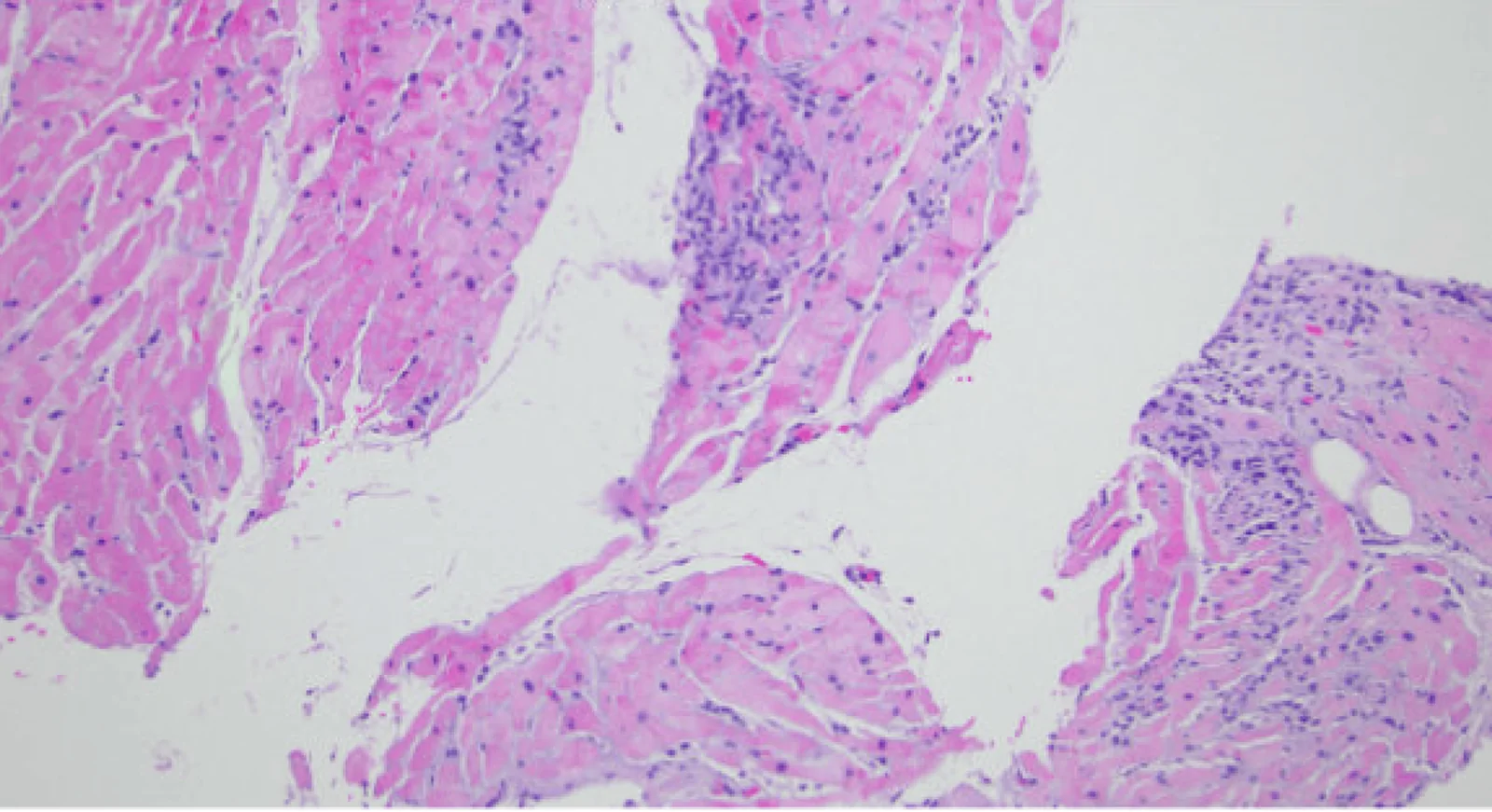
Endomyocardial biopsy demonstrating focal moderate lymphocytic and histiocytic myocarditis with associated myocyte necrosis; no giant cells, granulomas, or viral inclusions were identified

**Figure 6:**
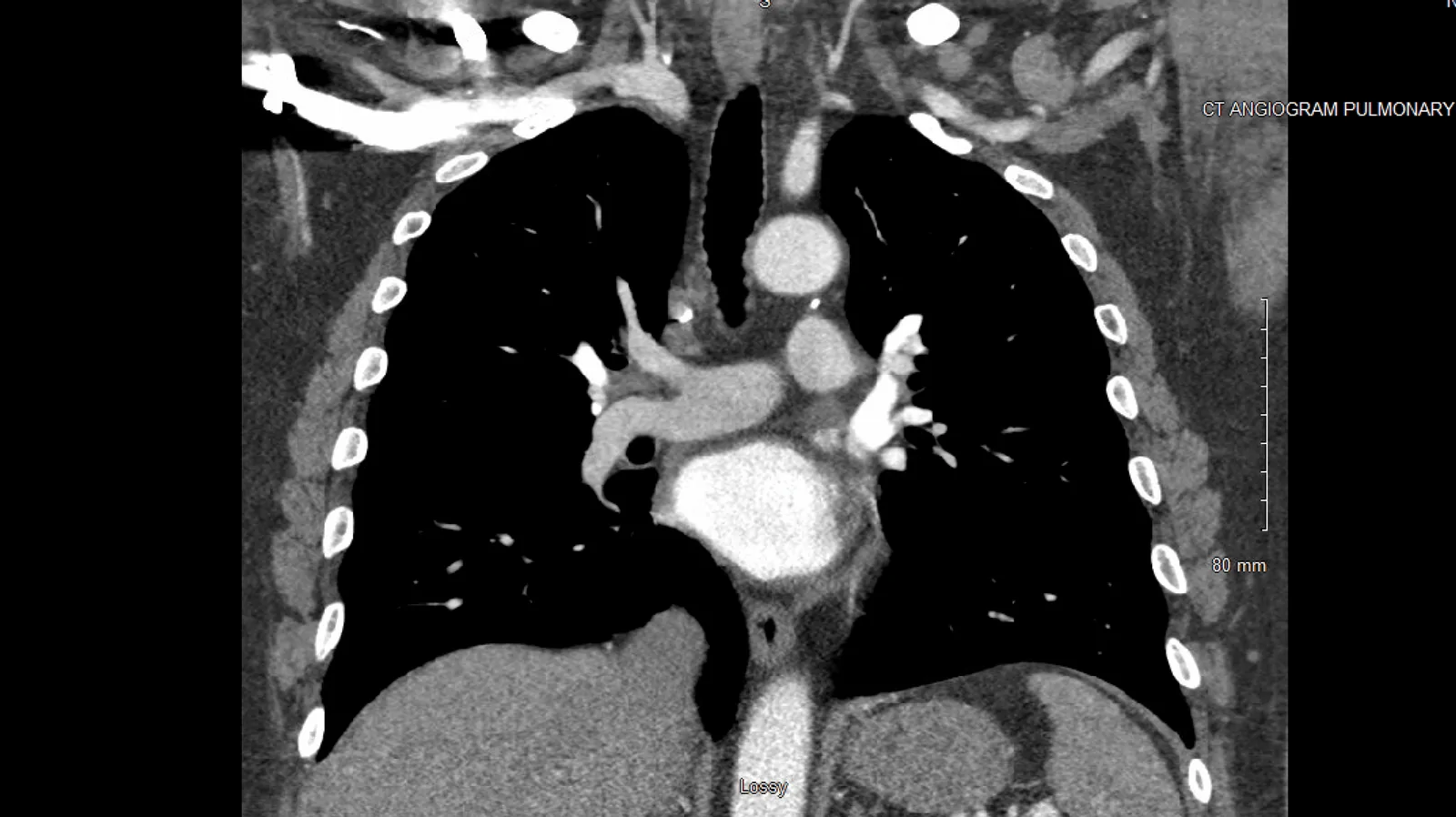
CT pulmonary angiography with no acute abnormalities

On the first day of steroid initiation, a myasthenia gravis antibody panel was ordered, along with electromyography (EMG)/nerve conduction study (NCS) and repetitive nerve stimulation studies. Acetylcholine receptor binding antibody returned negative at less than 0.30 nmol/L. Muscle-specific kinase antibody was also negative. Electromyography and nerve conduction studies showed chronic, mild C5-C6 radiculopathy changes, with minor inflammatory changes in the cervical paraspinal muscles. Repetitive nerve stimulation, performed at both slow and fast rates over the left abductor digiti minimi, showed no significant decremental or incremental response, providing no electrodiagnostic evidence of a neuromuscular junction transmission disorder. Negative inspiratory force and bedside spirometry monitored twice daily remained reassuring throughout the hospitalization.

The patient was advised to follow up with outpatient neurology for further evaluation of his unspecified neck weakness.

## Discussion

The patient's pretreatment cardiovascular risk factors included coronary artery disease, hypertension, and hyperlipidemia; functional status and baseline ECG prior to treatment initiation were normal. Baseline troponin, BNP/NT-proBNP, and echocardiographic assessment were not obtained prior to ICI initiation. This case supports considering baseline cardiac biomarker and echocardiographic assessment in patients with pre-existing cardiovascular risk factors prior to initiating potentially cardiotoxic systemic therapy. While the HFA-ICOS baseline cardiovascular risk stratification framework is well established for anthracycline- and HER2-targeted therapy, a validated, ICI-specific pretreatment risk stratification tool has not yet been established; though general guidelines support baseline cardiovascular risk assessment in this population.

This case illustrates a severe multisystem immune-related adverse event syndrome following a single cycle of combined enfortumab vedotin and pembrolizumab for Stage III urothelial carcinoma, with confirmed myocarditis and myositis, and probable immune-related hepatotoxicity.

Triple M syndrome describes the concurrent occurrence of immune checkpoint inhibitor-associated myocarditis, myositis, and myasthenia gravis, with reported mortality approaching 60%, thought to arise from shared antigenic targets in cardiac muscle, skeletal muscle, and at the neuromuscular junction. In this patient, myocarditis developed approximately three weeks after the first treatment cycle -- consistent with the recognized early time course of ICI-associated myocarditis. The diagnosis was confirmed histopathologically by endomyocardial biopsy demonstrating focal moderate lymphocytic myocarditis, and further supported by cardiac MRI demonstrating non-ischemic mid-myocardial late gadolinium enhancement in the basal inferolateral wall. Myositis was strongly supported by creatine phosphokinase 3,332 U/L, lactate dehydrogenase 730 U/L, myalgias, and cervical extensor weakness with drop head.

Acetylcholine receptor and muscle-specific kinase antibody testing, as well as repetitive nerve stimulation of the left abductor digiti minimi, were performed at the time of treatment onset rather than after a period of sustained immunosuppression. This suggests that their negative results are unlikely to be substantially confounded by treatment effect. While double-seronegative myasthenia gravis -- negative for both AChR and MuSK but potentially reactive to LRP4, agrin, or other less characterized antigens -- is a recognized entity [7], the absence of fatigable weakness on repeated examination, reassuring respiratory function parameters, and the early, unconfounded negative RNS and antibody results together make clinically significant myasthenia gravis unlikely in this case [8].

The discrepancy between the echocardiographic ejection fraction (55-60%) and CMR-derived ejection fraction (46%) may reflect limited echocardiographic windows and differing quantification methods rather than true physiologic change; the specific echocardiographic quantification method was not documented, limiting direct comparison with CMR-derived values. CMR's tissue characterization, however, uniquely identified late gadolinium enhancement consistent with myocarditis, a finding not assessable by echocardiography. The severely dilated left atrium (indexed volume 40 mL/m²) provided structural substrate for the atrial fibrillation that developed during admission. The biphasic troponin trajectory, with a post-procedural rise following percutaneous coronary intervention before a sustained downtrend, reflects the compounding effect of procedural myocardial injury superimposed on the underlying immune-mediated process.

Transaminases (AST/ALT) were elevated at presentation with a hepatocellular pattern, while alkaline phosphatase, GGT, total bilirubin, and INR remained within normal limits, arguing against a cholestatic or synthetic component. Given the concurrent myositis (creatine phosphokinase (CPK) 3,332 U/L), AST elevation alone could be attributable to muscle injury. However, ALT, which is more liver-specific, was also elevated and trended independently of CPK, suggesting a hepatic contribution.

Management followed established principles for severe immune checkpoint inhibitor-associated myocarditis: prompt drug discontinuation, high-dose corticosteroids, and intravenous immunoglobulin. The specific alternate-day dosing schedule for both agents reflected the treating team's individualized approach to this evolving multisystem presentation, rather than a standardized or protocol-defined interval. The concurrent Type C coronary lesion required percutaneous coronary intervention, adding complexity to anticoagulation management. The patient had no history of bleeding or hematuria, and the required DAPT therapy did not ultimately alter the course of his oncologic care.

New-onset atrial fibrillation in the context of severely dilated left atrium and active myocarditis was successfully managed with cardioversion and antiarrhythmic therapy. Previous studies have elucidated the association of arrhythmias in patients treated with ICIs; the most common being atrial fibrillation, followed by ventricular tachycardia, ventricular fibrillation, and atrioventricular (AV) block [9].

Both chemotherapeutic agents were permanently discontinued. Any future cancer-directed therapy will require careful risk-benefit assessment with preference for agents carrying lower immune-related toxicity profiles.

## Conclusions

This case demonstrates that combined enfortumab vedotin and pembrolizumab can precipitate severe, early-onset multisystem immune-related toxicity after a single cycle, requiring rapid recognition, prompt immunosuppression, and permanent discontinuation of therapy. Concurrent myocarditis and myositis raised suspicion for Triple M syndrome; however, the absence of clinical signs of myasthenia gravis and a negative repetitive nerve stimulation study argued against neuromuscular junction involvement, and Triple M syndrome was not confirmed.

Cardiac MRI is valuable for accurate characterization of immune checkpoint inhibitor-associated myocarditis and should be prioritized when suspicion persists. Immune-mediated myocardial injury may coexist with obstructive coronary artery disease, necessitating parallel management strategies. This case highlights the importance of systematic, evidence-based evaluation of overlapping immune-related adverse events before diagnostic labels are applied, and underscores the need for multidisciplinary collaboration in guiding decisions about ICI discontinuation and future oncologic management.
